# A Nomogram to Predict Benign/Malignant Mediastinal Lymph Nodes Based on EBUS Sonographic Features

**DOI:** 10.1155/2024/3711123

**Published:** 2024-02-29

**Authors:** Bingchao Ling, Weishun Xie, Yi Zhong, Taowen Feng, Yueli Huang, Lianying Ge, Aiqun Liu

**Affiliations:** Department of Endoscopy, Guangxi Medical University Cancer Hospital, Nanning 530021, China

## Abstract

**Background:**

Endobronchial ultrasound (EBUS) sonographic features help identify benign/malignant lymph nodes while conducting transbronchial needle aspiration (TBNA). This study aims to identify risk factors for malignancy based on EBUS sonographic features and to estimate the risk of malignancy in lymph nodes by constructing a nomogram.

**Methods:**

1082 lymph nodes from 625 patients were randomly enrolled in training (*n* = 760) and validation (*n* = 322) sets. The subgroup of EBUS-TBNA postoperative negative lymph nodes (*n* = 317) was randomly enrolled in a training (*n* = 224) set and a validation (*n* = 93) set. Logistic regression analysis was used to identify the EBUS features of malignant lymph nodes. A nomogram was formulated using the EBUS features in the training set and later validated in the validation set.

**Results:**

Multivariate analysis revealed that long-axis, short-axis, echogenicity, fusion, and central hilar structure (CHS) were the independent predictors of malignant lymph nodes. Based on these risk factors, a nomogram was constructed. Both the training and validation sets of 5 EBUS features nomogram showed good discrimination, with area under the curve values of 0.880 (sensitivity = 0.829 and specificity = 0.807) and 0.905 (sensitivity = 0.819 and specificity = 0.857). Subgroup multivariate analysis revealed that long-axis, echogenicity, and CHS were the independent predictors of malignancy outcomes of EBUS-TBNA postoperative negative lymph nodes. Based on these risk factors, a nomogram was constructed. Both the training and validation sets of 3 EBUS features nomogram showed good discrimination, with the area under the curve values of 0.890 (sensitivity = 0.882 and specificity = 0.786) and 0.834 (sensitivity = 0.930 and specificity = 0.636).

**Conclusions:**

Our novel scoring system based on two nomograms can be utilized to predict malignant lymph nodes.

## 1. Background

Many diseases involve the mediastinal lymph nodes, and the main causes are tuberculosis, nodal disease, inflammation, teratoma, thymoma, lung cancer, metastatic tumors, and lymphoma [1]. Identifying benign and malignant mediastinal lymph nodes is crucial to formulating treatment plans and determining the patient's prognosis [2, 3].

Many worldwide guidelines recommend EBUS-TBNA for staging lung cancer and diagnosing hilar and mediastinal lesions [4, 5]. EBUS-TBNA is also reported to have a higher diagnostic accuracy than computed positron emission tomography (PET) and computed tomography (CT) [6]. Patients undergoing EBUS-TBNA can be diagnosed with malignancy or benignity based on the sonographic features [7]. Hence, sonographic features of hilar and mediastinal lymph nodes have attracted increasing interest. In a retrospective assessment of 1061 lymph nodes, EBUS sonographic features such as shape, short-axis, echogenicity, margin, coagulation necrosis sign (CNS), and absence of CHS were widely used to identify benign or malignant mediastinal lymph nodes [8]. EBUS sonographic features are a useful tool to distinguish malignant or benign lymph nodes and can also be used to identify benign intrathoracic lymphadenopathy [9]. This study predicted tuberculous nodes from two sonographic features (the absence of clustered formation and the presence of necrosis signs) and two sonographic features of each category (absence of clustered formation, hilar perfusion or avascular, and CHS) predicted reactive lymphadenitis, as well as sarcoid nodes was predicted the optimal diagnostic efficiency by at least four sonographic features from five features (short-axis >1 cm, absence of CHS, nonhilar perfusion, margin, and clustered formation) [9]. In addition, EBUS sonographic features not only predict benign and malignant lymph nodes but also allow for further identification of EBUS-TBNA postoperative negative lymph nodes. A retrospective risk model study of lung cancer patients with negative EBUS-TBNA lymph nodes showed that heterogeneity was an important EBUS sonographic feature for predicting malignant lymph nodes [10]. However, to the best of our knowledge, no nomograms are currently available to predict the malignant lymph nodes and the risk stratification of lymph nodes deemed negative following EBUS-TBNA based on EBUS sonographic features.

The purpose of this study was to develop and validate a nomogram that accurately predicts the malignant lymph nodes and the risk stratification of lymph nodes deemed negative following EBUS-TBNA based on EBUS sonographic features.

## 2. Materials and Methods

### 2.1. Study Patients

Data for patients who underwent EBUS-TBNA due to unclear diagnosis of mediastinal enlarged lymph nodes in the Endoscopic Diagnosis Center of the Affiliated Cancer Hospital of Guangxi Medical University from February 2016 to June 2019 were analyzed retrospectively. Inclusion criteria included the following: (1) age 18 or older, (2) patients who underwent chest enhanced CT before EBUS-TBNA examination to assess the nature of mediastinal and hilar enlarged lymph nodes, (3) complete EBUS-TBNA and obtain lymph node tissue for histological and cytological examination, and (4) complete clinical and imaging data. Exclusion criteria included the following: (1) unable to obtain lymph node tissue by EBUS-TBNA, (2) loss of EBUS image or poor EBUS image quality, and (3) loss of follow-up or missing data.

Study ethics approval was granted by the Guangxi Medical University Cancer Hospital Ethical Review Committee (LW2023019). The patients provided written informed consent for the publication of their anonymized information in this article. This retrospective study was carried out in compliance with the STROBE guidelines [11].

### 2.2. EBUS-TBNA Procedure and Pathological Diagnosis

Patients were examined with a convex probe ultrasound bronchoscope (CP-EBUS; BF-UC260FW, Olympus, Tokyo, Japan) under moderate sedation with midazolam or propofol and local anesthesia with lidocaine. An ultrasound bronchoscope with a frequency of 10 MHz was used for scanning, and an ultrasound device (Eu-ME1 processor, Olympus) was used to generate images to record the sonographic features of lymph nodes. A dedicated 22-gauge needle (Olympus, NA-201XS-4022) was used for lymph node puncture. Each lymph node was punctured 2–5 times.

The tissue obtained by EBUS-TBNA was fixed in formalin, and the remaining aspirates were smeared on glass slides and fixed with 95% ethanol. Finally, the treated specimens were submitted for examination. Any positive histology or cytology of the puncture specimen was judged to be positive. The final diagnosis of malignant lymph nodes was determined by EBUS-TBNA's malignant cytological and/or histological findings or surgical and pathological confirmation. Postoperative pathological results of EBUS-TBNA were benign, but the imaging findings were highly suspected of malignant lesions. The samples were obtained in other ways and confirmed by pathological examination. If the abovementioned methods still fail to rule out malignant lesions, radiological and clinical follow-up will be carried out for at least 6 months.

### 2.3. EBUS Image Categories

**Figure 1:**
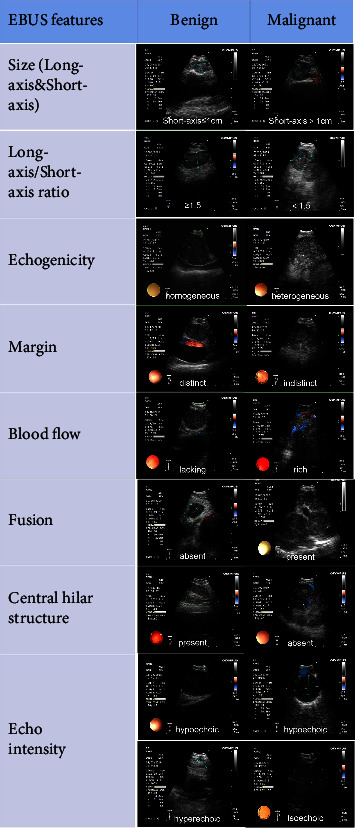
Representative morphology of EBUS features.

We evaluated ultrasound features according to the following nine categories (Figure 1) [12]: long-axis (cm), short-axis (>1 cm or <1 cm), long-axis/short-axis ratio (<1.5 or ≥1.5), echogenicity (heterogeneous or homogeneous), margin (distinct or indistinct), blood flow (rich or lacking), fusion (absent or present), CHS (absent or present), and echo intensity (hypoechoic or isoechoic or hyperechoic). Echo intensity was defined as hypoechoic, isoechoic, and hyperechoic contrasted with the surrounding tissue. Heterogeneous echogenicity was defined as several small areas of varying echogenicity, but do not contain major vascular structures. Distinct margin was defined as more than half of the margin was visible. Fusion was defined as multiple lymph nodes fused into a single lymph node station. CHS was defined as a linear, flat, hyperechoic region in the center of the lymph node. Blood flow was defined as rich and lacking, with lacking suggesting grades 0-1, whereas rich suggesting grades 2-3.

A comparison of the EBUS feature of each lymph node with the final diagnosis was conducted to determine the predictive accuracy of malignant lymph nodes.

### 2.4. Statistical Analyses

Descriptive statistics were reported as frequencies with percentages or interquartile ranges (ranges). The training and validation sets were randomly grouped in a 7 : 3 ratio. Comparison of training and validation sets was performed by using the Mann–Whitney *U* test (continuous variables) and the chi-square test (categorical variables). Univariate and multivariate analyses of EBUS features predicting the accuracy of malignant lymph nodes were performed by using logistic regression models. A nomogram for predicting the malignant lymph nodes and the risk stratification of the EBUS-TBNA postoperative negative lymph nodes were developed by using a logistic regression model. Nomogram's accuracy in predicting was evaluated by using the receiver operating characteristic (ROC) curve and the area under the curve (AUC). Calibration curves were used for evaluating the goodness of fit of the nomogram. Decision curve analysis (DCA) and clinical impact curve (CIC) were conducted to estimate the net clinical benefits. Statistics were considered significant at a *P* value < 0.05 (two-sided).

The statistical analysis was performed with R software (version 4.1.3). The logistic regression analysis, nomogram construction plots, and nomogram calibration plots were used by the “rms” package. The DCA and CIC were performed using the “rmda” package. A ROC curve analysis was conducted using the “pROC” package.

## 3. Results

### 3.1. Patients and Lymph Nodes

**Figure 2:**
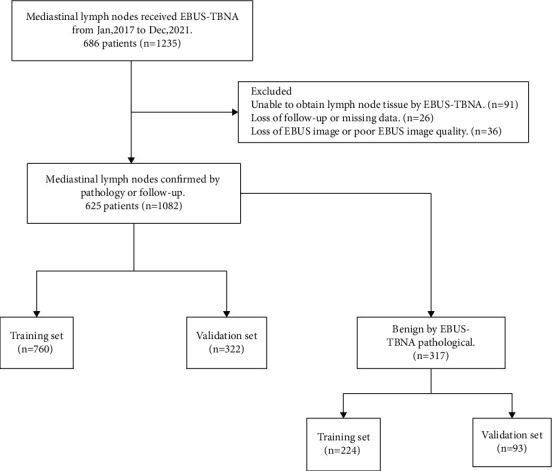
Flowchart of nomogram development and verification.

In total, 686 patients (1235 lesions) underwent EBUS-TBNA. 61 patients (153 lesions) were excluded due to the inability to obtain lymph node tissue, loss of follow-up or missing data, and loss of EBUS image or poor EBUS image quality. About 1082 lesions of 625 patients were analyzed. A flowchart illustrating the recruitment of lymph nodes and patients is shown in Figure 2.

A summary of the patient's clinical characteristics is shown in Table 1, including gender (female (198/625, 31.7%) and male (427/625, 68.3%)), age (59 years, median), smoking index (20.00, median), family history of cancer (absent (557/625, 89.1%) and present (68/625, 10.9%)), and previous disease (171/625, 27.4%). Ultrasound image features of all lymph nodes are summarized in Table 2, including long-axis (2 cm, median), short-axis (>1 cm (882/1082, 81.5%) and ≤1 cm (200/1082, 18.5%)), long-axis/short-axis ratio (≥1.5 (229/1082, 21.2%) and <1.5 (853/1082, 78.8%)), number of passes per node (3, median), echo intensity (hyperechoic (32/1082, 3%), hypoechoic (1041/1082, 96.2%), and isoechoic (9/1082, 0.8%)), echogenicity (heterogeneous (671/1082, 62%) and homogeneous (411/1082, 38%)), margin (indistinct (866/1082, 80%) and distinct (216/1082, 20%)), blood flow (rich (890/1082, 82.3%) and lacking (192/1082, 17.7%)), fusion (absent (822/1082, 76%) and present (260/1082, 24%)), and CHS (absent (740/1082, 68.4%) and present (342/1082, 31.6%)). Supplementary Table 1 shows the proportion of each station. Unclear diagnosis of mediastinal enlarged lymph nodes was frequently observed in the stations 4R and 7 lymph nodes (625/1082, 57.8%).

The pathological diagnosis of each lymph node is shown in Table 3. EBUS-TBNA pathology diagnosed 765 lymph nodes as malignant and 317 lymph nodes as benign. The final diagnosis report diagnosed 838 lymph nodes as malignant and 244 lymph nodes as benign.

### 3.2. Developing and Validating a Nomogram to Predict Malignant Lymph Nodes

1082 lymph nodes were randomly divided into a training set (*n* = 760) and a validation set (*n* = 322) in a 7 : 3 ratio. The ultrasound image features within the training and validation sets did not differ significantly, except for the number of passes per node (*P*=0.017) (Table 4).

**Figure 3:**
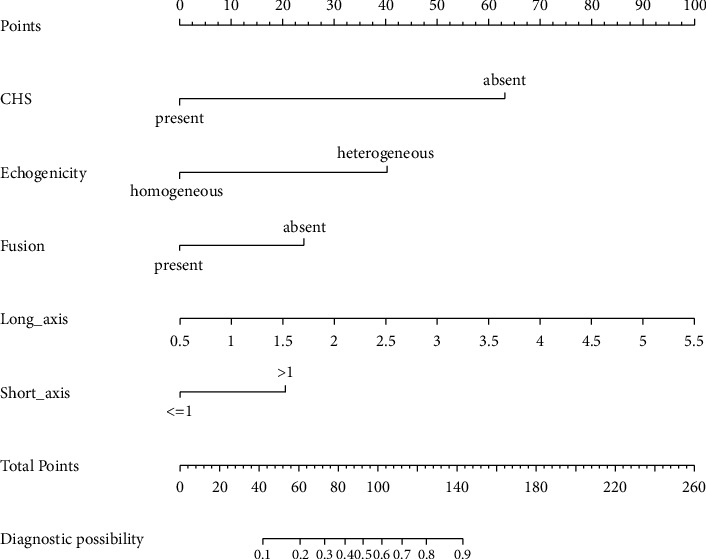
Developed 5 EBUS features nomogram with the following features: long-axis, short-axis, echogenicity, fusion, and CHS. CHS, central hilar structure.

A summary of the results of the univariate and multivariate logistic regression analyses of the ultrasound image features in the training set is shown in Table 5. In the univariate analysis, smoking index (*P*=0.01), long-axis (*P* < 0.001), short-axis (*P* < 0.001), echogenicity (*P* < 0.001), blood flow (*P*=0.002), fusion (*P*=0.002), and CHS (*P* < 0.001) were associated with a malignant lymph node. In the multivariate analysis, long-axis (*P* < 0.001), short-axis (*P*=0.005), echogenicity (*P* < 0.001), fusion (*P*=0.002), and CHS (*P* < 0.001) were the independent impact factors of malignancy outcomes. According to these features, 5 EBUS features nomogram was constructed (Figure 3). Prediction of malignancy outcomes could be obtained by summing each point (the total points).

**Figure 4:**
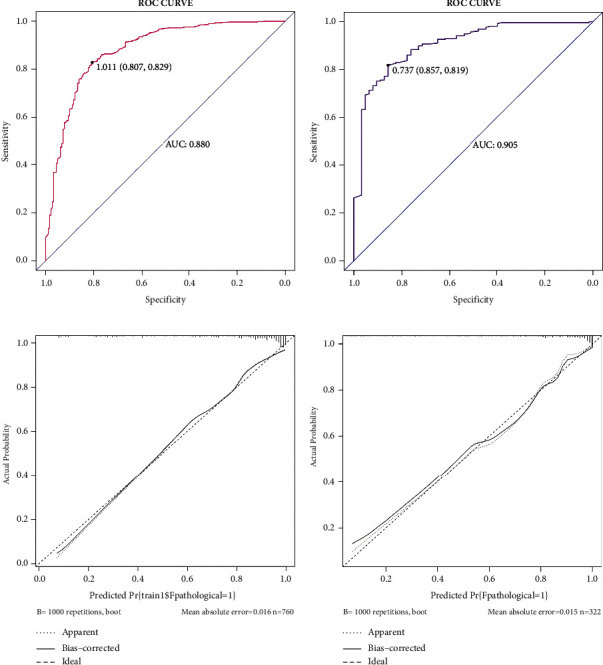
The performance of 5 EBUS features nomogram in the training set and the validation set. (a) ROC curve of 5 EBUS features nomogram for predicting malignant lymph nodes in the training dataset. (b) ROC curve of 5 EBUS features nomogram for predicting malignant lymph nodes in the validation dataset. (c) Calibration curve of 5 EBUS features nomogram for predicting malignant lymph nodes in the training dataset. (d) Calibration curve of 5 EBUS features nomogram for predicting malignant lymph nodes in the validation dataset.

**Figure 5:**
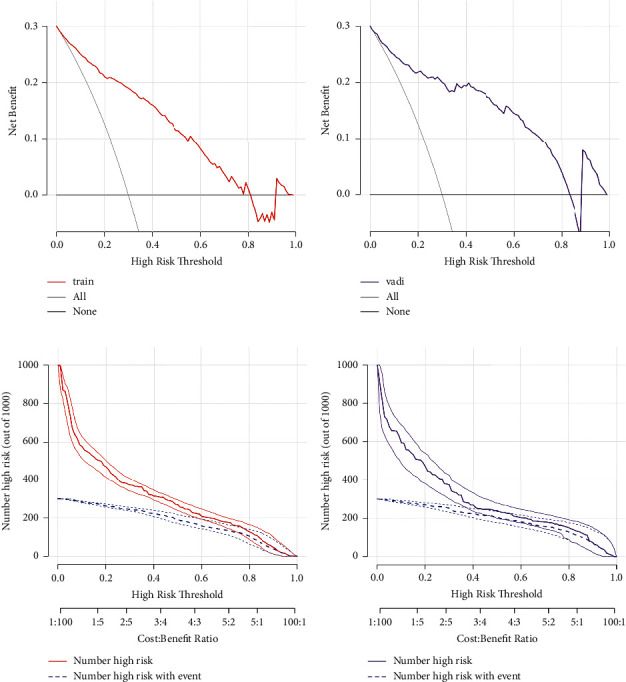
DCA and CIC of 5 EBUS features nomogram in the training set and the validation set. (a) DCA of 5 EBUS features nomogram for predicting malignant lymph nodes in the training set. (b) DCA of 5 EBUS features nomogram for predicting malignant lymph nodes in the validation set. (c) CIC of 5 EBUS features nomogram for predicting malignant lymph nodes in the training set. (d) CIC of 5 EBUS features nomogram for predicting malignant lymph nodes in the validation set. CIC, clinical impact curve; DCA, decision curve analysis.

Both the training and validation sets of the 5 EBUS features nomogram were accurate in predicting malignancy outcomes (Figures 4(a) and 4(b)). This nomogram had an AUC of 0.880 (sensitivity = 0.829 and specificity = 0.807) in the training set and 0.905 (sensitivity = 0.819, specificity = 0.857) in the validation set. In addition, the calibration plots of the 5 EBUS features nomogram showed good agreement between predicted and actual malignancy outcomes in training and validation sets (Figures 4(c) and 4(d)). The DCA shows that the 5 EBUS features nomogram had a good predictive efficiency in the training set and validation sets (Figures 5(a) and 5(b)). The high-risk threshold of the training set was approximately 0–0.8 and that of the validation set was approximately 0–0.85, which was the most beneficial for the prediction of malignant lymph nodes. The CICs were established based on the 5 EBUS features nomogram DCA to help us more intuitively comprehend their substantial value (Figures 5(c) and 5(d)).

The nomogram also demonstrated strong predictive capabilities for various cancer cell types, including lung cancer and non-lung cancer lymph nodes. Notably, it achieved a high accuracy in predicting the diagnostic yield, as evidenced by AUC values of 0.750 (sensitivity = 0.809 and specificity = 0.609) for lung cancer lymph nodes and 0.698 (sensitivity = 0.744 and specificity = 0.533) for non-lung cancer lymph nodes. Furthermore, the nomogram exhibits favorable performance in predicting the diagnostic rates of different pathologic types of lung cancer. Specifically, the AUC values for adenocarcinoma, squamous carcinoma, small cell carcinoma, and other lung cancers were 0.669 (sensitivity = 0.531 and specificity = 0.814), 0.729 (sensitivity = 0.867 and specificity = 0.600), 0.742 (sensitivity = 0.632 and specificity = 0.760), and 0.682 (sensitivity = 0.767 and specificity = 0.594), respectively (Supplementary Table 2).

### 3.3. Developing and Validating a Nomogram to Predict Malignancy Outcomes of the EBUS-TBNA Postoperative Negative Lymph Nodes

317 EBUS-TBNA postoperative negative lymph nodes were randomly divided into a training set (*n* = 224) and a validation set (*n* = 93) in a 7 : 3 ratio. The ultrasound image features within the training and validation sets did not differ significantly, except for the echo intensity (*P*=0.033) (Table 6).

**Figure 6:**
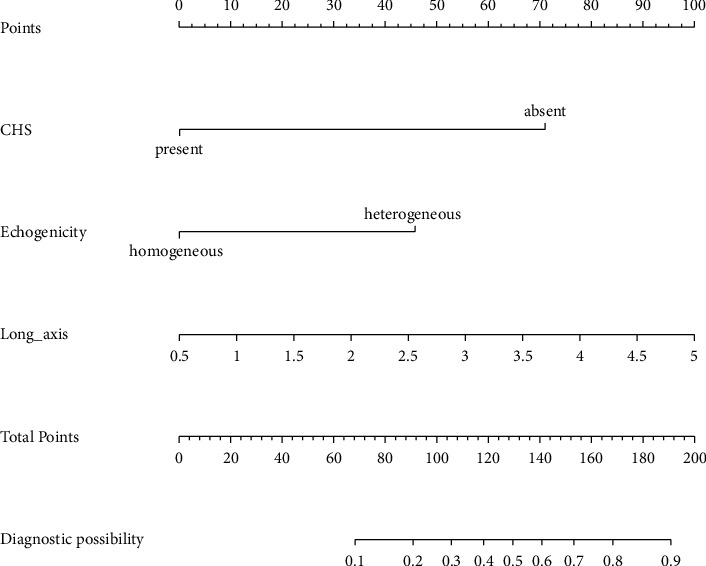
Developed a 3 EBUS features nomogram with the following features: long-axis, echogenicity, and CHS. CHS, central hilar structure.

A summary of the results of the univariate and multivariate logistic regression analyses of the ultrasound image features in the training set is shown in Table 7. In the univariate analysis, long-axis (*P* < 0.001), short-axis (*P* < 0.001), number of passes per node (*P*=0.032), echogenicity (*P* < 0.001), margin (*P*=0.042), fusion (*P*=0.001), and CHS (*P* < 0.001) were associated with malignancy outcomes in the EBUS-TBNA postoperative negative lymph nodes. In the multivariate analysis, long-axis (*P* < 0.001), echogenicity (*P* < 0.001), and CHS (*P* < 0.001) were the independent impact factors of malignancy outcomes in the EBUS-TBNA postoperative negative lymph nodes. According to these features, 3 EBUS features nomogram was constructed (Figure 6). A prediction of malignancy outcomes in the EBUS-TBNA postoperative negative lymph nodes can be obtained by summing each point (the total points).

**Figure 7:**
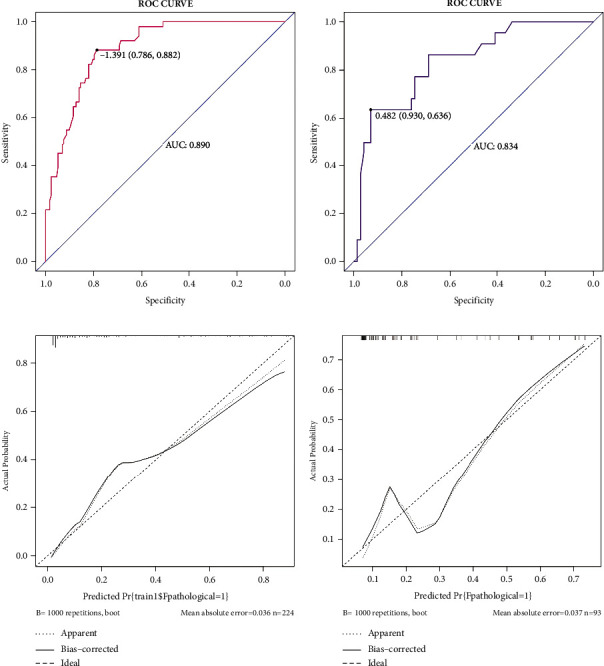
The performance of 3 EBUS features nomogram in the training set and the validation set. (a) ROC curve of 3 EBUS features nomogram for predicting the malignancy outcomes of the EBUS-TBNA postoperative negative lymph nodes in the training dataset. (b) ROC curve of 3 EBUS features nomogram for predicting the malignancy outcomes of the EBUS-TBNA postoperative negative lymph nodes in the validation dataset. (c) Calibration curve of 3 EBUS features nomogram for predicting the malignancy outcomes of the EBUS-TBNA postoperative negative lymph nodes in the training dataset. (d) Calibration curve of 3 EBUS features nomogram for predicting the malignancy outcomes of the EBUS-TBNA postoperative negative lymph nodes in the validation dataset.

**Figure 8:**
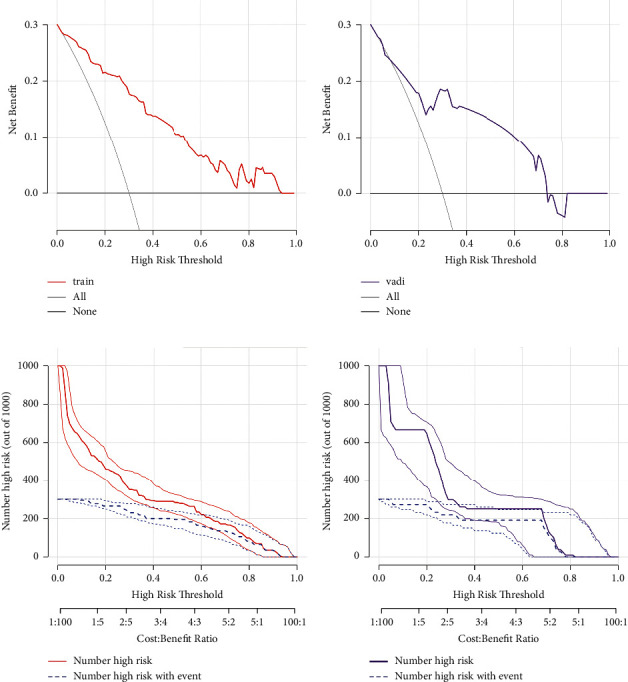
DCA and CIC of 3 EBUS features nomogram in the training set and the validation set. (a) DCA of 3 EBUS features nomogram for predicting the malignancy outcomes of the EBUS-TBNA postoperative negative lymph nodes in the training set. (b) DCA of 3 EBUS features nomogram for predicting the malignancy outcomes of the EBUS-TBNA postoperative negative lymph nodes in the validation set. (c) CIC of 3 EBUS features nomogram for the malignancy outcomes of the EBUS-TBNA postoperative negative lymph nodes in the training set. (d) CIC of 3 EBUS features nomogram for the malignancy outcomes of the EBUS-TBNA postoperative negative lymph nodes in the validation set. CIC, clinical impact curve; DCA, decision curve analysis.

Both the training and validation sets of the 3 EBUS features nomogram were accurate in predicting malignancy outcomes (Figures 7(a) and 7(b)). This nomogram had an AUC of 0.890 (sensitivity = 0.882 and specificity = 0.786) in the training set and 0.834 (sensitivity = 0.930 and specificity = 0.636) in the validation set. In addition, calibration plots of the 3 EBUS features nomogram showed a good agreement between predicted and actual malignancy outcomes in training and validation sets (Figures 7(c) and 7(d)). The DCA shows that the 3 EBUS features nomogram had a good predictive efficiency in the training set and validation sets (Figures 8(a) and 8(b)). The high-risk threshold of the training set was approximately 0–0.9 and that of the validation set was approximately 0.08–0.75, which was the most beneficial for the prediction of malignancy outcomes in the EBUS-TBNA postoperative negative lymph nodes. The CICs were established based on the 3 EBUS features nomogram DCA to help us more intuitively comprehend their substantial value (Figures 8(c) and 8(d)). 73.5% of the EBUS-TBNA postoperative negative lymph nodes were classified as other benign lymph nodes, and it was also important to recognize malignant outcomes in these lymph nodes. Supplementary Table 3 shows that the nomogram accurately predicted other benign lymph nodes with an AUC of 0.874 (sensitivity = 0.847 and specificity = 0.783).

## 4. Discussion

In this study, we successfully established a systematic scoring model based on two nomograms to distinguish benign/malignant lymph nodes and EBUS-TBNA postoperative negative lymph nodes. In predicting malignant lymph nodes, our 5 EBUS features nomogram consisted of long-axis, short-axis, echogenicity, fusion, and CHS. The optimal AUC value for this nomogram was 0.905 which was better than the Canada LN score (AUC = 0.72) and eight EBUS features (AUC = 0.857) [13, 14] and also had a good predictive efficacy in predicting various cancer cell types. In predicting malignancy outcomes of EBUS-TBNA postoperative negative lymph nodes, our 3 EBUS features nomogram consisted of long-axis, echogenicity, and CHS. The optimal AUC value for this nomogram was 0.89, which was of high sensitivity and specificity. It was the first nomogram that predicted the malignancy outcomes of EBUS-TBNA postoperative negative lymph nodes only based on EBUS features [10].

Several studies have evaluated the diagnostic performance of each EBUS feature and scoring model based on EBUS features for predicting malignant lymph nodes [8, 13–19]. Among these studies, Fujiwara et al. were the first to report on EBUS features for predicting malignant lymph nodes [8]. 487 patients and 1061 lymph nodes were analyzed retrospectively. A distinct margin, round shape, heterogeneous echogenicity, and coagulation necrosis sign were independent predictors of metastasis in multivariate analysis, each with an OR of 3.05, 3.1, 1.96, and 5.64. Morishita et al. reported on multi-EBUS features [14]. A total of 597 lymph nodes were evaluated retrospectively from 302 patients. Among a multivariate analysis of metastasis risk, short-axis (>1 cm), absence of CHS, heterogeneous echogenicity, presence of CNS, and blue-dominant images were the most predictive factors, with odds ratios of 1.86, 1.901, 20.4, 3.86, and 3.46. In addition, Morishita et al. drew ROC curves based on the results of multivariate analysis, eight EBUS features, and six B-mode features, with AUC values of 0.894, 0.857, and 0.84. Diagnostic parameters of EBUS features were different in each study. Our results show that the absence of CHS (OR = 13.11) and heterogeneous echogenicity (OR = 5.46) have a strong ability to predict malignant lymph nodes compared with the remaining EBUS features. Several studies have found similar trends [14, 15]. In addition, long-axis, short-axis (>1 cm), and absence of fusion were also found to be associated with predicting malignant lymph nodes in our study. Interestingly, there were few studies on long-axis and fusion. Only Wang et al. reported that the long-axis (>1.67 cm) was more accurate at predicting malignant lymph nodes than the short-axis [19]. Our study also showed a similar result. The long-axis (>1.67 cm) had a higher diagnostic accuracy for predicting malignant lymph nodes than the short-axis (>1 cm) in our 5 EBUS features nomogram (Figure 3). Since there were few studies on the long-axis, the optimal cut-off value of the long-axis was controversial, so we analyzed the long-axis as a continuous variable. The absence of fusion was an independent predictive factor of malignant lymph nodes in our study. However, this outcome was contrary to that of Wang et al. who found that the presentation of fusion was an independent predictive factor of malignant lymph nodes [19]. These outcomes must be interpreted with caution because the presence of fusion could be seen in both benign and malignant diseases [20].

Few previous studies have focused on EBUS-TBNA postoperative negative lymph nodes, and only Evison et al. investigated a risk stratification model to categorise EBUS-TBNA postoperative negative lymph nodes based on EBUS, CT, and PET [10]. This retrospective study included 329 lymph nodes. Lymph node SUV, the SUV ratio, and heterogeneous echogenicity were independently predictive of malignancy in EBUS-TBNA postoperative negative lymph nodes. The only heterogeneous echogenicity was the EBUS feature. Our study found that long-axis, heterogeneous echogenicity, and absence of CHS were the independent predictors of malignancy. The long-axis and absence of CHS were a unique finding that had not been reported earlier in predicting the malignancy outcomes of EBUS-TBNA postoperative negative lymph nodes. Several studies have shown that long-axis and absence of CHS were significantly correlated with malignant lymph nodes [8, 14, 19, 21], so our study was a particularly useful finding.

Clinical applications of EBUS features require certain criteria. Researchers developed some scoring systems to explore the best cut-offs by combining several features. Wang et al. developed a scoring system based on nonhilar perfusion, presence of matting, absence of CHS, and round shape, with a diagnostic accuracy range of 24.57–82.68% and at least two of the features could achieve the best performance in predicting malignancy [19]. Hylton et al. developed a 4-point score: margins, short-axis diameter, necrosis, and central hilar structure [13]. Scoring ≥3 suggests biopsy. The AUC of this model was 0.72, which was of high sensitivity and specificity. Compared with the previous studies [13, 19], our two nomograms displayed excellent performance. Both nomograms showed a good predictive ability (AUC = 0.905 and 0.89), a good diagnostic accuracy (the highest accuracy of both was 90%), and a high clinical net benefit (DCA and CIC analysis) in predicting malignant lymph nodes. The good predictive performance and ease of use made these two nomograms easy to formulate strategies in the real world.

This study has some limitations. First, the study was retrospective and conducted at a single center, so it may have suffered from a selection bias. In addition, the sample size for predicting malignancy outcomes of EBUS-TBNA postoperative negative lymph nodes was small, which could have affected the credibility of this study. Hence, an external validation with multicenters and larger samples might be the best option. Furthermore, patients from diverse backgrounds participated in this study. Different benign and malignant diseases, for example, lymphoma, and granulomatous inflammation usually show different EBUS patterns [9, 20]. As a result, their results may differ.

## 5. Conclusions

Evaluation of lymph nodes with EBUS sonographic features would predict malignant lymph nodes and malignancy outcomes in the EBUS-TBNA postoperative negative lymph nodes. Our novel scoring system using the 5 EBUS features nomogram (long-axis, short-axis, echogenicity, fusion, and CHS) and 3 EBUS features nomogram (long-axis, echogenicity, and CHS) is useful for predicting malignant lymph nodes.

## Figures and Tables

**Table 1:** Clinical characteristics.

Characteristics	Overall (*n* = 625)
Gender	
Male	427 (68.3%)
Female	198 (31.7%)
Age (median (IQR))	59.00 [52.00, 66.00]
Smoking index (median (IQR))	20.00 [0.00, 600.00]
Family history of cancer	
Absent	557 (89.1%)
Present	68 (10.9%)
Previous disease	
Hypertension	120
Diabetes	36
Coronary heart disease	7
COPD	8

**Table 2:** Ultrasound image features of all lymph nodes.

Characteristics	Overall (*n* = 1082)
Long-axis (median [IQR])	2.00 [1.50, 2.77]
Short-axis	
>1 cm	882 (81.5%)
≤1 cm	200 (18.5%)
Long-axis/short-axis ratio	
≥1.5	229 (21.2%)
<1.5	853 (78.8%)
Number of passes per node (median [IQR])	3.0 [2.0, 3.0]
Echo intensity	
Hyperechoic	32 (3.0%)
Hypoechoic	1041 (96.2%)
Isoechoic	9 (0.8%)
Echogenicity	
Heterogeneous	671 (62%)
Homogeneous	411 (38%)
Margin	
Indistinct	866 (80%)
Distinct	216 (20%)
Blood flow	
Rich	890 (82.3%)
Lacking	192 (17.7%)
Fusion	
Absent	822 (76.0%)
Present	260 (24.0%)
Central hilar structure	
Absent	740 (68.4%)
Present	342 (31.6%)

**Table 3:** Pathological diagnosis of all lymph nodes.

Pathological type	EBUS-TBNA pathology	Final pathology
Malignant	765	838
Adenocarcinoma	523	563
Squamous cell carcinoma	86	93
SCLC	77	76
NSCLC-NOS	36	42
Metastatic tumor	28	32
Neuroendocrine tumor	9	11
Adenosquamous carcinoma	0	7
Lymphoma	0	6
Thymoma	2	3
Large cell lung cancer	2	2
Lymphoepitheliomatoid carcinoma	1	2
Yolk cystic tumor	1	1
Benign	317	244
Tuberculosis	24	44
Lymphadenitis	40	40
Granulomatous inflammation	17	19
*Aspergillus* infection	0	3
Schwannoma	2	2
Cryptococcal infection	0	2
Cyst	1	1
Other benign lymph node	233	133

**Table 4:** Ultrasound image features for predicting malignant lymph nodes in the training and validation sets.

Characteristics	Total (*n* = 1082)	Training set (*n* = 760)	Validation set (*n* = 322)	*P* value
Long-axis (median [IQR])	2.000 [1.500, 2.770]	2.000 [1.500, 2.752]	2.000 [1.555, 2.800]	0.602
Number of passes per node (median [IQR])	3.000 [2.000, 3.000]	3.000 [2.000, 3.000]	3.000 [2.000, 3.000]	0.017
Short-axis (%)				0.390
>1 cm	882 (81.5%)	614 (80.8%)	268 (83.2%)	
≤1 cm	200 (18.5%)	146 (19.2%)	54 (16.8%)	
Long-axis/short-axis ratio (%)				0.955
≥1.5	229 (21.2%)	160 (21.1%)	69 (21.4%)	
<1.5	853 (78.8%)	600 (78.9%)	253 (78.6%)	
Echo intensity (%)				0.087
Hyperechoic	32 (3.0%)	25 (3.3%)	7 (2.2%)	
Hypoechoic	1041 (96.2%)	726 (95.5%)	315 (97.8%)	
Isoechoic	9 (0.8%)	9 (1.2%)	0 (0.00)	
Echogenicity (%)				0.980
Heterogeneous	671 (62.0%)	472 (62.1%)	199 (61.8%)	
Homogeneous	411 (38.0%)	288 (37.9%)	123 (38.2%)	
Margin (%)				0.712
Indistinct	866 (80.0%)	611 (80.4%)	255 (79.2%)	
Distinct	216 (20.0%)	149 (19.6%)	67 (20.8%)	
Blood flow (%)				0.646
Rich	890 (82.3%)	622 (81.8%)	268 (83.2%)	
Lacking	192 (17.7%)	138 (18.2%)	54 (16.8%)	
Fusion (%)				0.268
Absent	822 (76.0%)	585 (77.0%)	237 (73.6%)	
Present	260 (24.0%)	175 (23.0%)	85 (26.4%)	
Central hilar structure (CHS) (%)				0.237
Absent	740 (68.4%)	511 (67.2%)	229 (71.1%)	
Present	342 (31.6%)	249 (32.8%)	93 (28.9%)	

**Table 5:** Univariate and multivariate logistic regression analyses of the training set in the whole cohort.

Characteristics	Univariate		Multivariate	
	OR (95% CI)	*P* value	OR (95% CI)	*P* value
Long-axis	2.87 (2.21–3.72)	<0.001	2.33 (1.63–3.35)	<0.001
Short-axis				
≤1	Reference			
>1	4.73 (3.21–6.96)	<0.001	2.38 (1.3–4.35)	0.005
Long-axis/short-axis ratio				
<1.5	Reference			
≥1.5	0.79 (0.53–1.17)	0.239		
Number of passes per node	1.12 (0.94–1.32)	0.198		
Echo intensity				
Hypoechoic	Reference			
Hyperechoic	1.05 (0.38–2.90)	0.919		
Isoechoic	0.41 (0.09–1.87)	0.250		
Echogenicity				
Homogeneous	Reference			
Heterogeneous	5.51 (3.83–7.93)	<0.001	5.65 (3.6–8.85)	<0.001
Margin				
Indistinct	Reference			
Distinct	1.29 (0.84–2.00)	0.249		
Blood flow				
Lacking	Reference			
Rich	1.89 (1.27–2.81)	0.002	1.57 (0.93–2.66)	
Fusion				
Absent	Reference			
Present	2.02 (1.29–3.14)	0.002	0.36 (0.19–0.68)	0.002
Central hilar structure				
Present	Reference			
Absent	9.44 (6.46–13.78)	<0.001	15.59 (9.06–26.83)	<0.001

**Table 6:** Ultrasound image features for predicting the malignancy outcomes of lymph nodes deemed negative following EBUS-TBNA in the training and validation sets.

Characteristics	Total (*n* = 317)	Training set (*n* = 224)	Validation set (*n* = 93)	*P* value
Long-axis (median [IQR])	1.590 [1.300, 2.100]	1.600 [1.295, 2.185]	1.500 [1.300, 2.000]	0.395
Number of passes per node (median [IQR])	3.000 [2.000, 3.000]	3.000 [2.000, 3.000]	3.000 [2.000, 3.000]	0.184
Short-axis (%)				0.597
>1 cm	203 (64.0%)	146 (65.2%)	57 (61.3%)	
≤1 cm	114 (36.0%)	78 (34.8%)	36 (38.7%)	
Long-axis/short-axis ratio (%)				1.000
≥1.5	77 (24.3%)	54 (24.1%)	23 (24.7%)	
<1.5	240 (75.7%)	170 (75.9%)	70 (75.3%)	
Echo intensity (%)				0.033
Hyperechoic	11 (3.5%)	4 (1.8%)	7 (7.5%)	
Hypoechoic	298 (94.0%)	215 (96.0%)	83 (89.3%)	
Isoechoic	8 (2.5%)	5 (2.2%)	3 (3.2%)	
Echogenicity (%)				0.720
Heterogeneous	123 (38.8%)	85 (38.0%)	38 (40.9%)	
Homogeneous	194 (61.2%)	139 (62.0%)	55 (59.1%)	
Margin (%)				0.498
Indistinct	264 (83.3%)	184 (82.1%)	80 (86.0%)	
Distinct	53 (16.7%)	40 (17.9%)	13 (14.0%)	
Blood flow (%)				1.000
Rich	243 (76.7%)	172 (76.8%)	71 (76.3%)	
Lacking	74 (23.3%)	52 (23.2%)	22 (23.7%)	
Fusion (%)				0.616
Absent	256 (80.8%)	183 (81.7%)	73 (78.5%)	
Present	61 (19.2%)	41 (18.3%)	20 (21.5%)	
Central hilar structure (CHS) (%)				0.442
Absent	131 (41.3%)	89 (39.7%)	42 (45.2%)	
Present	186 (58.7%)	135 (60.3%)	51 (54.8%)	

**Table 7:** Univariate and multivariate logistic regression analyses of the training set for the EBUS-TBNA diagnosed benign cohort.

Characteristics	Univariate		Multivariate	
	OR (95% CI)	*P* value	OR (95% CI)	*P* value
Long-axis	2.3 (1.55–3.41)	<0.001	2.05 (1.14–3.7)	0.017
Short-axis				
≤1	Reference			
>1	2.66 (1.25–5.65)	0.011	1.2 (0.4–3.61)	0.740
Long-axis/short-axis ratio				
<1.5	Reference			
≥1.5	0.83 (0.39–1.76)	0.63		
Number of passes per node	1.39 (1.03–1.87)	0.032	1.0 (0.67–1.48)	0.998
Echo intensity				
Hypoechoic	Reference			
Hyperechoic	1.16 (0.12–11.4)	0.899		
Isoechoic	2.32 (0.38–14.28)	0.364		
Echogenicity				
Homogeneous	Reference			
Heterogeneous	6.07 (3.05–12.07)	<0.001	5.46 (2.33–12.78)	<0.001
Margin				
Indistinct	Reference			
Distinct	0.32 (0.11–0.96)	0.042	0.28 (0.08–1.04)	0.057
Blood flow				
Lacking	Reference			
Rich	1.54 (0.69–3.43)	0.286		
Fusion				
Absent	Reference			
Present	3.56 (1.73–7.33)	0.001	1.07 (0.39–2.93)	0.902
Central hilar structure				
Present	Reference			
Absent	14.84 (6.49–33.92)	<0.001	13.11 (4.74–36.27)	<0.001

## Data Availability

The datasets produced and analyzed during this study are not publicly available due to our research center's policy, but can be requested from the corresponding author.

## Abbreviations

AUC: Area under the curve
CIC: Clinical impact curve
CT: Computed tomography
CHS: Central hilar structure
CNS: Coagulation necrosis sign
COPD: Chronic obstructive pulmonary disease
DCA: Decision curve analysis
EBUS-TBNA: Endobronchial ultrasound-guided transbronchial needle aspiration
EBUS: Endobronchial ultrasound
NSCLC-NOS: Non-small cell lung cancer-not otherwise specified
PET: Positron emission tomography
ROC: Receiver operating characteristic
SCLC: Small cell lung cancer.

## Supplementary Materials

Supplementary Materials Supplementary Table 1: the puncture site of the lymph node. Supplementary Table 2: the 5 EBUS features nomogram predicts diagnostic yield of malignant lymph nodes. Supplementary Table 3: the 3 EBUS features nomogram predicts diagnostic yield of other benign lymph node.
