# Supplementary material for: A Nomogram to Predict Benign/Malignant Mediastinal Lymph Nodes Based on EBUS Sonographic Features
Source: Int J Clin Pract. 2024 Feb 29;2024:3711123. doi: 10.1155/2024/3711123 (PMC10919979; doi:10.1155/2024/3711123)
Supplement: Supplementary Materials — Supplementary Table 1: the puncture site of the lymph node. Supplementary Table 2: the 5 EBUS features nomogram predicts diagnostic yield of malignant lymph nodes. Supplementary Table 3: the 3 EBUS features nomogram predicts diagnostic yield of other benign lymph node. [file 3711123.f1.docx]

| **Supplementary Table 1**  The puncture site of the lymph node. | |
| --- | --- |
| Node | Count |
| 2L | 4 |
| 2R | 45 |
| 4L | 80 |
| 4R | 345 |
| 7 | 280 |
| 8 | 4^*^ |
| 10L | 103 |
| 10R | 177 |
| 11L | 15 |
| 11R | 12 |
| 12L | 5 |
| 12R | 11 |
| 13R | 1 |

^*^ The 4 LNs were sampled by endobronchial ultrasound-guided transesophageal needle aspiration (EBUS-TENA) .

| **Supplementary Table 2** The 5 EBUS features nomogram predicts diagnostic yield of malignant lymph nodes. | | | |
| --- | --- | --- | --- |
| Pathological type | sensitivity | specificity | AUC |
| Lung cancer | 0.809 | 0.609 | 0.750 |
| Adenocarcinoma | 0.531 | 0.814 | 0.669 |
| Squamous cell carcinoma | 0.867 | 0.600 | 0.729 |
| SCLC | 0.632 | 0.760 | 0.742 |
| Other lung cancer^*^ | 0.767 | 0.594 | 0.682 |
| Non-lung cancer^#^ | 0.744 | 0.533 | 0.698 |

^*^Other lung cancer: NSCLC-NOS, Neuroendocrine tumor, Adenosquamous carcinoma, large cell lung cancer and Lymphoepitheliomatoid carcinoma.

^#^ Non-lung cancer: metastatic tumor, Lymphoma, Thymoma, Yolk cystic tumor.

| **Supplementary Table 3** The 3 EBUS features nomogram predicts diagnostic yield of other benign lymph node. | | | |
| --- | --- | --- | --- |
| Pathological type | sensitivity | specificity | AUC |
| Other benign lymph node | 0.847 | 0.783 | 0.874 |
